# Intraocular lymphoma masquerading as unilateral hypopyon anterior uveitis: a case report

**DOI:** 10.1186/s12348-022-00302-5

**Published:** 2022-07-28

**Authors:** Ghodsieh Zamani, Atefeh Hajipour, Babak Ganjeifar, Nazanin Ebrahimiadib, Seyedeh Maryam Hosseini

**Affiliations:** 1 Eye Research Center, Mashhad University of Medical Sciences, Mashhad, Iran; 2 Department of Neurosurgery, Mashhad University of Medical Sciences, Mashhad, Iran; 3 Ophthalmology Department, Retina Service, Farabi Eye Hospital, Tehran University of Medical Sciences, Tehran, Iran; 4 Ophthalmology Department, Ocular Immunology and Uveitis Foundation, Waltham, MA USA; 5 Eye Research Center, Khatam-al-Anbia Eye Hospital, Qarani Blvd, Mashhad, 9195965919 Iran

**Keywords:** Vitreoretinal lymphoma, Diffuse large B-cell lymphoma, Masquerade syndrome, Hypopyon uveitis, Anterior uveitis

## Abstract

**Purpose:**

To report an unusual case of unilateral anterior segment large B-cell intraocular lymphoma (IOL) presenting as a recurrent hypopyon anterior uveitis.

**Case presentation:**

A 55-year-old female was referred because of recurrent unilateral anterior hypopyon uveitis with partial response to topical corticosteroid. All of the laboratory tests, review of systems and ocular sampling results were unremarkable. Given a high concern for masquerades syndromes, cytological specimens were obtained 3 times and the last sample showed large B cell lymphoma. First, it appeared confined to the eye and initially responded favorably to local chemotherapy (methotrexate and rituximab) but later went on to develop systemic involvement. CNS lymphoma was detected on the third brain MRI 6 months following ocular involvement. At this time, systemic chemotherapy was started. In the last 18 months’ follow-up, visual acuity was 20/30 in the right eye without posterior segment or fellow eye involvement.

**Conclusion:**

Unusual presentation of intraocular lymphoma as a unilateral isolated anterior hypopyon uveitis should be kept in mind. This report emphasizes the importance of precise work-ups and multiple ocular biopsies to confirm the diagnosis of intraocular lymphoma.

## Introduction

Vitreoretinal lymphoma is a rare form of non-Hodgkin lymphoma that occurs in the lymphoid tissues of the eye (ie, retina, vitreous, sub–retinal pigment epithelium (sub- RPE), and optic nerve head [1]. The disease is considered vitreoretinal lymphoma if at the time of diagnosis, it is limited to the eye and there is no intracranial involvement [2]. However, up to 80% of patients presenting with vitreoretinal lymphoma will eventually manifest intracranial malignancy [3]. Diagnosis of vitreoretinal lymphoma remains a challenge as it can mimic chronic posterior uveitis. The most common clinical signs and symptoms include floaters, painless moderate loss of vision, moderate vitritis, RPE pigmentary changes and sub-retinal infiltrate [4, 5].

Herein, we report a case of vitreoretinal lymphoma presenting with unusual hypopyon anterior uveitis in the absence of vitritis.

## Case presentation

**Fig. 1:**
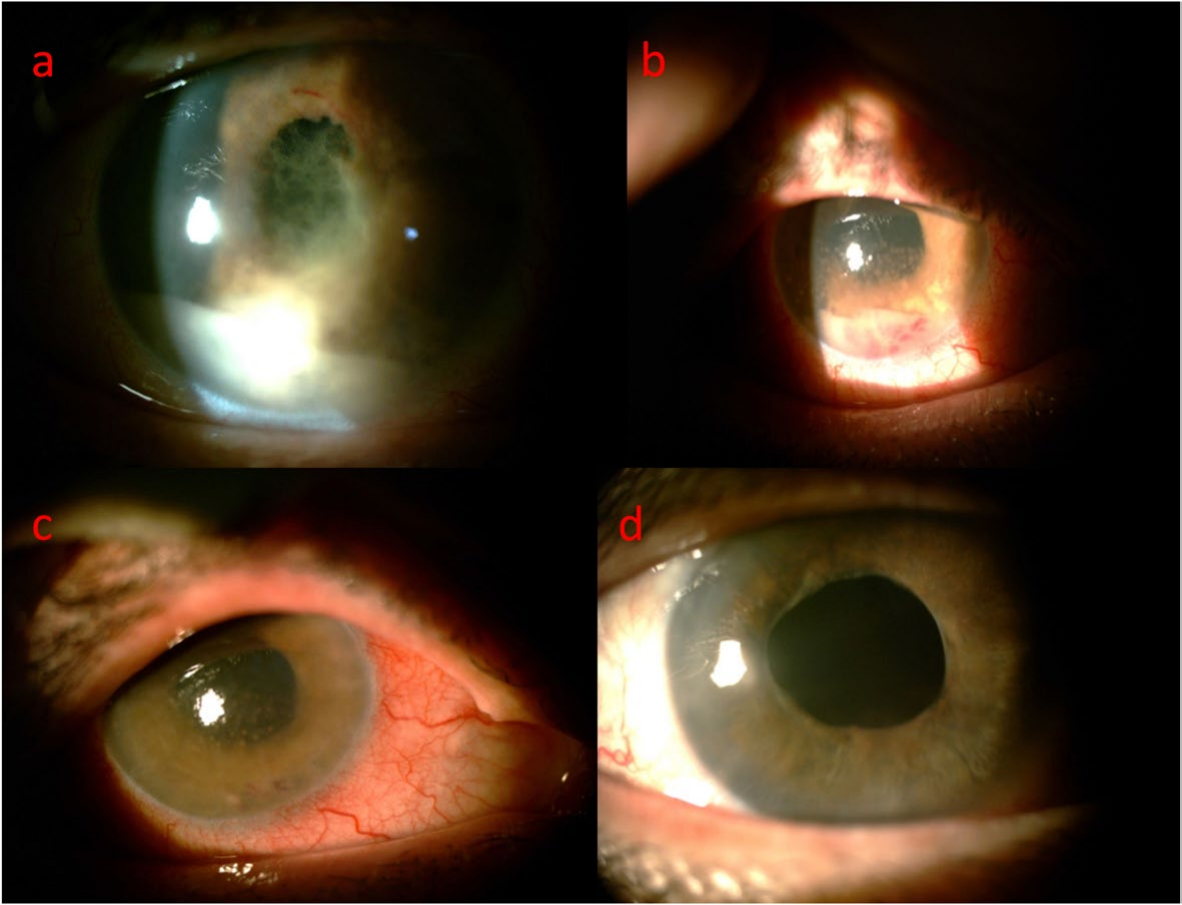
Slit photography of the right eye; hypopyon anterior uveitis with severe fibrinous reaction following 2 months therapy as a uveitis case (**a**), Following third ocular sampling and cataract surgery (**b**), two weeks following intraocular methotrexate injection (**c**), One year after the first presentation (**d**)

**Fig. 2:**
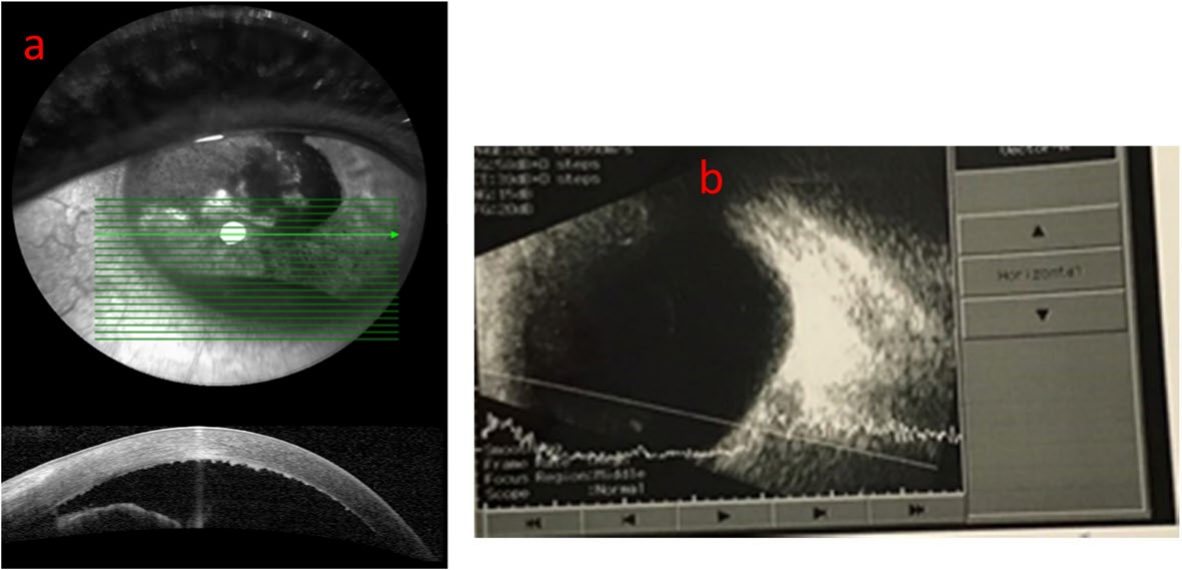
**a** Anterior segment OCT shows thick hyper-reflective material on the corneal endothelium, elevation and hyper-reflectivity of the iris surface with a beneath hypo-reflectivity. **b** B scan ultrasonography of the right eye shows no vitreous involvement despite severe hypopyon anterior uveitis precluding fundus exam at first presentation

**Fig. 3:**
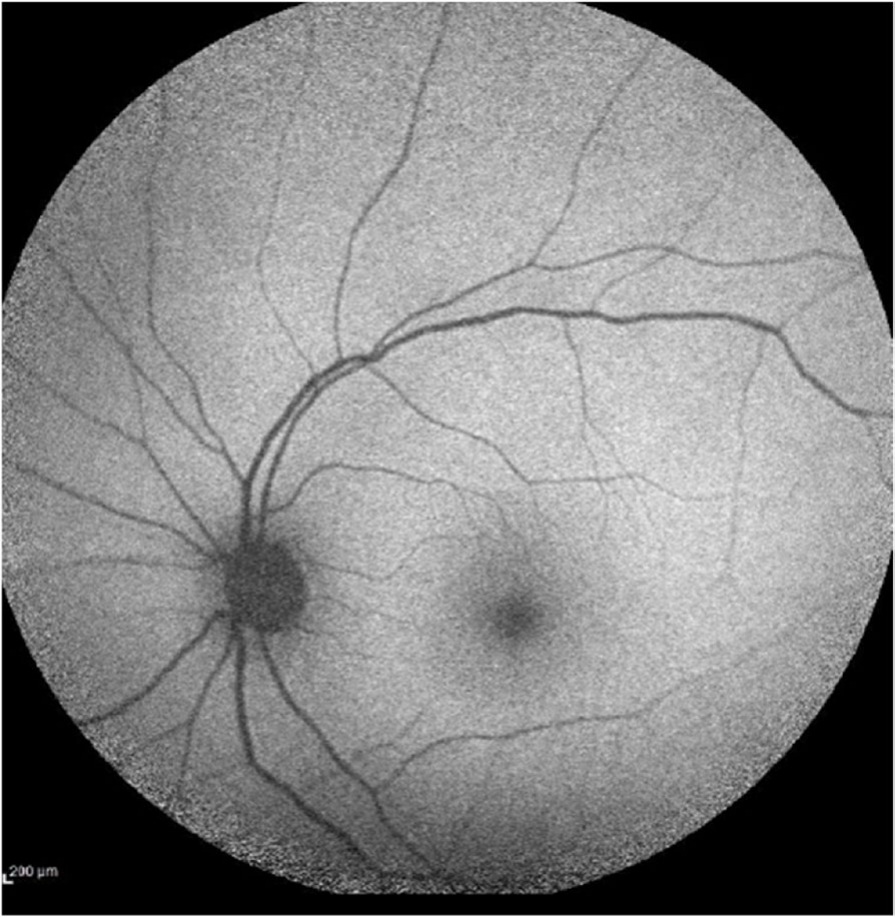
Normal fundus autofluorescence (FAF) of the left eye at first presentation. FAF of the right eye c not be obtained because of hazy media

**Fig. 4:**
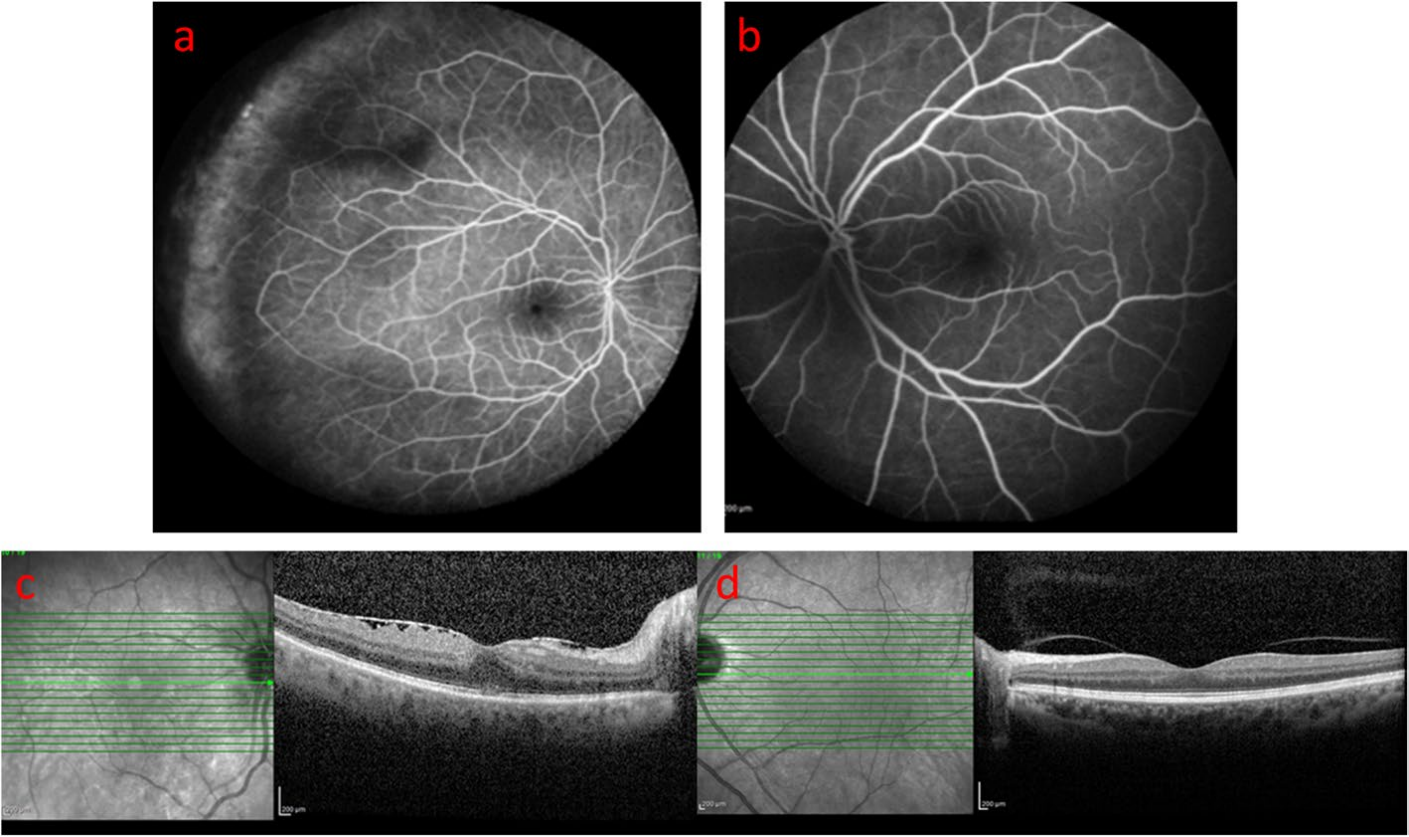
Six-month after intra-ocular treatment: Fluorescein angiography of the right (**a**) and the left eye (**b**); Macular OCT of the right eye (**c**) disclosed epiretinal membrane and normal choroid and normal left eye (**d**)

A 55-year-old female had been referred to uveitis clinic of Khatam-Al-Anbia eye hospital for refractory, relapsing unilateral hypopyon anterior uveitis (AU). She complained of decreased visual acuity in her right eye since 2 months ago associated with redness and ocular pain. The past medical, ocular, drug and familial history were unremarkable. At the first examination best corrected visual acuity (BCVA) was 20/32 and 20/20 in the right and left eye, respectively. Relative afferent pupillary defect was negative. Intraocular pressure was normal in both eyes. In slit lamp examination of the right eye, non-mutton fat keratic precipitates (KPs) and 0.2 mm hypopyon had been observed (Fig. 1a). Iris was bulged inferonasaly (Fig. 2a). Fundus examination and imaging were normal without any vitreous involvement (Fig. 2b, Fig. 4 a). The left eye examination was normal (Figs. 3, 4b).

Regarding to hypopyon AU, rheumatologic consult was done for excluding Behcet disease and ankylosing spondylitis and other rheumatologic diseases. Laboratory tests including complete blood count (CBC, diff), erythrocyte sedimentation rate (ESR), C-reactive protein (CRP), HLA B27, HLA B5, liver and kidney function tests, HIV and hepatitis serology, PPD test, peripheral blood smear and blood and urine culture for exclusion of endogenous endophthalmitis were performed. All tests and work ups got back normal. Given the presentation of inflammation as serosanguinous, granulomatous features and inconclusive initial workups we assumed that herpes viruses may be the etiology of refractory uveitis. With the possible diagnosis of herpetic anterior uveitis, topical corticosteroid, cycloplegic and oral acyclovir treatment was prescribed without significant response.

Due to worsening of visual acuity (VA), persistence of anterior uveitis and iris bulging despite treatment with topical and systemic corticosteroid, systemic antiviral and antibiotics (gemifloxacin and azithromycin), anterior chamber (AC) sampling for polymerase chain reaction (PCR), cytologic and microbial evaluation was performed with suspicious of infectious and masquerades etiologies that were inconclusive. Systemic investigation for masquerade syndromes including oncology consultation for accurate systemic examinations, CBC diff, peripheral blood smear, brain MRI, lung and abdomen CT scans and ultrasonography was also unremarkable. One month later, VA decreased to 2/100. In her examination 1 mm serosanguinous reaction was seen in AC and fundus was not visible. The left eye was normal again. There was no vitreous opacity in B-Scan of the right eye (Fig. 2 b).

Again, with suspicion of intraocular lymphoma, we evaluated the patient for systemic lymphoma and leukemia. Brain and orbital magnetic resonance imaging (MRI), lumbar puncture and oncology consultation were requested. The results of all evaluations were negative. Second cytologic evaluation of AC sampling was negative for atypical cells, too.

Progressive decreasing VA to hand motion with increasing hypopyon without any response to anti-microbial treatment including virus, bacteria and fungi convinced us for third cytologic evaluation. Finally, cataract surgery without lens implantation associated with AC sampling for cytology and flow cytometry was performed with guidance of ocular pathologist. This sample confirmed large B cell lymphoma and it was positive for CD19 and CD20.

With the diagnosis of intraocular lymphoma which surprisingly affects only the anterior chamber, intravitreal methotrexate (MTX) 0.4 mg/0.1 cc weekly for one month and then monthly for 5 months and rituximab 1 mg/0.1 cc monthly for 2 month (regarding to the posterior capsule opening) were injected to the right eye. Significant response was observed after 2 weeks with disappearance of hypopyon, KPs and improvement of BCVA to 20/40 (Figs. 1 and 4c).

**Fig. 5:**
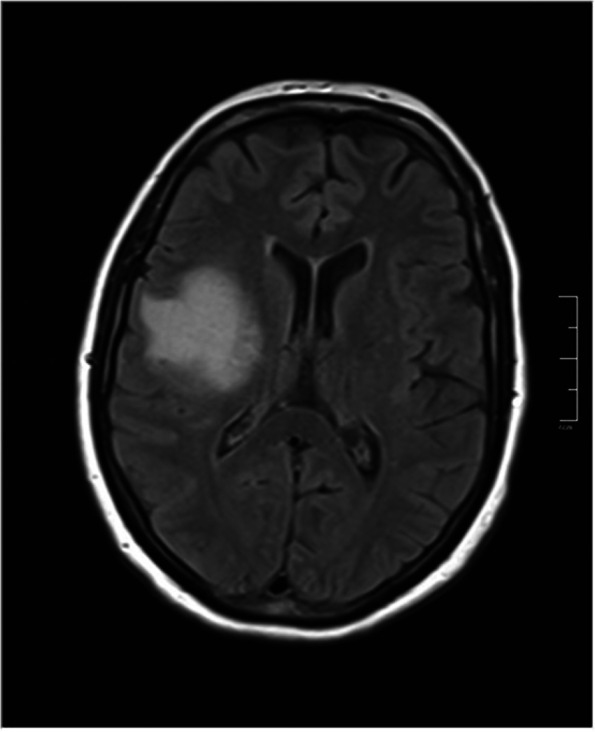
Second brain MRI (FLAIR sequence) showed hyper-intense lesion involving periventricular and subcortical white matter

Six months after first presentation, second brain and orbital MRI revealed abnormal signal areas with increased enhancement (Fig. 5). In head and neck ultrasonography, 5 mm benign lymph node in her left axilla and 4 mm benign lymph node around right and left carotid artery was detected. After 1 month she complained of generalized tremor. According to hematologist and oncologist consult, systemic chemotherapy and radiotherapy was performed. In the last 18 months follow up, BCVA was 20/30 and the eye was quiet without posterior segment or fellow eye involvement with stable general condition (Fig. 1d).

## Discussion

The clinical findings detailed in this case provide an atypical presentation and evolution of intraocular lymphoma. Non-Hodgkin’s lymphoma involves ocular tissues either as a primary tumor or as secondary metastasis from systemic disease. Although primary intraocular lymphoma cells have been identified in the optic nerve, ciliary body, and iris of a small number of patients by histopathology, these sites of infiltration have rarely been observed on clinical examination. Velez G et al. described two patients with iris infiltration by primary intraocular lymphoma [6]. Pseudohypopyon is one of the signs of recurrent vitreoretinal lymphoma. Lobo A et al. described a patient with a rare and unusual presentation of hypopyon uveitis who was eventually diagnosed with diffuse large B-cell lymphoma (DLBCL) [7]. Papaliodis GN et al. reported that a patient with a previous medical history of peripheral B-cell lymphoma developed hypopyon 3 months after R-CHOP chemotherapy and prophylactic intrathecal chemotherapy [8]. In both cases, the combination of systemic chemotherapy and radiation was effective to cause the remission of pseudohypopyon. In cytological analysis, pseudohypopyon cells are medium to large pleomorphic lymphoid cells with irregular hyperchromatic nuclei and prominent mitotic activity. The phenotypes of these cells were found to be partly positive for CD20 [7, 8]. Kitao et al. reported a 64-year-old woman with pseudohypopyon as one of the signs of vitreoretinal lymphoma recurrency. Although pseudohypopyon was temporarily controlled with intravitreal MTX, this treatment did not completely induce its remission [9].

The majority of vitreoretinal lymphomas are of a DLBCL histologic subtype, though occasionally T-cell lymphomas can occur. They usually express CD19, CD20, CD22, PAX5, BOB.1, and OCT2 [10].

Our patient was a challenging and interesting case because the initial manifestation of intraocular lymphoma was hypopyon. However, this sign is one of the rare signs of intraocular lymphoma especially as a presenting sign. Furthermore, despite the clinical suspicion of lymphoma, the histology of the sample was negative for malignant cells twice. It is probably due to fragility of lymphoma cells and their tendency to rupture and degenerate quickly, so we must alert pathologist that the samples are on their way. Cytological examination of rapidly transported, unfixed vitreous specimens is considered the gold standard in exclusion of intraocular lymphoma in patients with idiopathic steroid resistant chronic uveitis. These specimens are difficult to interpret, and reports of “false negatives” or “false positives” are common. Coupland SE et al. demonstrated that cytomorphology and immunoreactivity of vitreous specimens are well preserved following HOPE (Herpes-glutamic acid buffer mediated Organic solvent Protection Effect) fixation. This fixation appears to be promising in simplifying the transportation of vitreous specimens in patients with masquerade syndrome and may improve the diagnostic reliability of these specimens [11].

Another surprising point in this case is sparing of posterior segment and unilaterality during the 18 months’ follow-up that is unusual for intraocular lymphoma. Given the course of our patient, we propose that pseudo-hypopyon with unilateral and isolated anterior segment involvement is a rare clinical presentation of intraocular B-cell lymphoma. A high index of suspicion along with insisting on multiple intraocular fluid analysis are essential to establish the diagnosis. Clinicians should be aware of this dramatic mode of presentation, which is unusual for ocular lymphoma.

## Data Availability

The datasets used during the current report are available from the corresponding author on reasonable request.

## Abbreviations

CNS: Central nervous system
DLBCL: Diffuse large B-cell lymphomas
BCVA: Best-corrected visual acuity
PCR: Polymerase chain reaction
AU: Anterior uveitis
AC: Anterior chamber
MRI: Magnetic resonance imaging
RPE: Retinal pigment epithelium
